# Ageing Signatures and Disturbed Muscle Regeneration in Muscle Proteome of Inclusion Body Myositis

**DOI:** 10.1002/jcsm.13845

**Published:** 2025-06-08

**Authors:** Geert M. de Vries, Bob Asselbergh, Alice Monticelli, Peter De Jonghe, Stuart Maudsley, Peter Y. K. Van Den Bergh, Anne Bigot, Jan L. De Bleecker, Biljana Ermanoska, Willem De Ridder, Jonathan Baets

**Affiliations:** ^1^ Translational Neurosciences and Peripheral Neuropathy Group University of Antwerp Antwerp Belgium; ^2^ Laboratory of Neuromuscular Pathology Institute Born‐Bunge, University of Antwerp Antwerp Belgium; ^3^ Histology and Cellular Imaging, Neuromics Support Facility VIB Center for Molecular Neurology, VIB Antwerp Belgium; ^4^ Department of Biomedical Sciences University of Antwerp Antwerp Belgium; ^5^ Department of Neurology, Neuromuscular Reference Centre Antwerp University Hospital Antwerp Belgium; ^6^ Receptor Biology Lab, Department of Biomedical Sciences University of Antwerp Antwerp Belgium; ^7^ Department of Neurology University Hospital St‐Luc, University of Louvain Brussels Belgium; ^8^ Sorbonne Université, Inserm, Institut de Myologie, Centre de Recherche en Myologie Paris France; ^9^ Laboratory for Neuropathology, Division of Neurology Ghent University Hospital Ghent Belgium

**Keywords:** idiopathic inflammatory myopathy, KDM5A, muscle regeneration, proteomics

## Abstract

**Background:**

Inclusion body myositis (IBM) is the most common acquired myopathy in adults over the age of 50 years, characterised by inflammatory and degenerative features that lead to progressive muscle weakness and physical disability for lack of effective therapies. The complex interplay between inflammatory and degenerative processes, occurring seemingly simultaneously, presents a challenge to systematically dissect disease pathology and discover novel therapeutic targets.

**Methods:**

To identify proteomic IBM disease signatures and upstream regulators of disease processes in an unbiased manner, we performed high‐resolution iTRAQ‐labelled mass spectrometry on whole muscle lysates of 28 IBM patients and 28 control individuals. Validation experiments were carried out by conducting immunohistochemical (IHC) stainings on KDM5A and myogenin using control and IBM patient muscle tissue sections. Human myoblasts were used to study involvement of KDM5A, a selected candidate‐upstream regulator, in IBM pathomechanisms in vitro.

**Results:**

A total of 627 significantly differentially expressed proteins were found in IBM patients compared to control individuals. The proteomics dataset strongly reflected inflammatory signatures, dysregulations in cellular energy metabolism and altered myogenesis in IBM muscle. Identification of upstream regulators of IBM pathology yielded KDM5A as the top activated and RB1 as the top inhibited upstream regulator. KDM5A, a histone demethylase involved in transcription regulation and (myogenic) differentiation, interacts with RB1 and interconnects core IBM disease signatures in patient muscle tissue. IHC stainings on muscle tissue showed increased presence of myogenin‐positive myonuclei (*p* < 0.0001). KDM5A levels were increased in these myogenin‐positive myonuclei in IBM patient muscle tissue compared to healthy controls (*p* < 0.0001). In vitro differentiation of myoblasts showed gradual KDM5A downregulation throughout myogenic differentiation, confirming presence in immature myoblasts and low levels in more mature myotubes. Proof‐of‐concept pharmacological inhibition of KDM5A with ryuvidine showed a significant effect on amyloid precursor protein (APP) abundance (*p* = 0.0003) and aggregation (*p* = 0.0132) in a conditional IBM‐mimicking inflammatory model.

**Conclusions:**

This unbiased proteomics study reflects known core features of IBM pathomechanisms while simultaneously providing novel insights into the proteomic landscape of IBM, most notably dysregulation of metabolic pathways and failure of myogenesis. The identification and exploration of KDM5A as a potential upstream driver of disease pathology could interconnect failure of myogenic differentiation with (known) disease processes in IBM and provides a target for future study and therapy.

## 1 Introduction

Inclusion body myositis (IBM) is the most common acquired myopathy among adults over 50 years of age and is classified as an idiopathic inflammatory myopathy (IIM) [1]. IBM differs clinically from other IIMs, particularly due to its slow progressive nature and the strikingly selective and often asymmetric involvement of specific distal muscle groups [2]. While IIMs typically respond well to immune suppression, clinical trials investigating immune‐modulating drugs in IBM were negative [3, 4]. The refractory nature of IBM and phenotypical overlap with other IIMs complicates the accurate and timely diagnosis, resulting in a high medical burden for IBM patients, with chronically debilitating muscle wasting [5, 6].

Diagnostic criteria comprise of late age of onset, characteristic clinical features, including weakness of deep finger flexors and selective weakness of the quadriceps, and histopathological findings [6]. Muscle biopsies of IBM patients typically show concomitant chronic inflammatory changes, with endomysial infiltration of immune cells and MHC‐I upregulation, degenerative features with cytoplasmic deposits of aggregated/misfolded hallmark proteins (SQSTM1/p62, amyloid precursor protein [APP] and TDP43) and mitochondrial abnormalities [1]. Though IBM is recently considered rather a primary inflammatory disorder, the combined phenotypical features of chronic inflammation and cell‐intrinsic degeneration continue to puzzle both clinicians and researchers on the exact primary disease nature [5, 6, 7, 8].

This phenotypic duality of IBM also complicates the accurate modelling of the full spectrum of IBM downstream pathology, with established cellular models commonly tailored to study either primary inflammatory or degenerative hypothesis [6, 9, 10]. The current disease models cover the majority of the classical IBM hallmarks and have been successfully employed to identify loss of proteostasis as a potentially common downstream pathway implicated in IBM pathology [9, 11].

While a number of recent small‐scale studies dissecting the transcriptomes and proteomes from IBM patients have advanced the field, the molecular triggers of pathology remain unknown [12, 13, 14, 15, 16]. In this study, we used proteomic profiling of muscle tissue derived from IBM patients, with the aim to (1) identify dysregulated protein candidates that might be upstream of IBM pathology and (2) manipulate these candidates in IBM cellular models to explore their potential to modify disease signatures.

## 2 Methods

### 2.1 Patients and Muscle Biopsies

Twenty‐eight IBM patients (12 women, 16 men, mean age 69.6 ± 8.5 years at the time of biopsy, range 55–85 years) of whom freshly frozen muscle tissue was available were included. Clinical and myopathological details were documented, assuring homogeneity of IBM muscle specimens. Diagnosis of IBM was based on the 2013 ENMC criteria, upon re‐evaluation also fulfilling the 2024 ENMC criteria [17]. Twenty‐eight control individuals, biopsied for subjective myalgia but without clinical, morphological or electrophysiological abnormalities, were matched based on biopsied muscle, sex and age (12 women, 16 men, age 63.1 ± 11.0 years at the time of biopsy, range 49–88 years). Muscle biopsies were initially performed as part of routine diagnostics and were critically reassessed for the purpose of this study. Biopsies were obtained from quadriceps, tibialis anterior or deltoid muscles and were analysed with standard histological and immunohistochemistry (IHC) light microscopy. The complete set of muscle specimens was used for proteomic analyses and further focused IHC experiments were performed on a selection of muscle biopsies. For all IBM patients, endomysial inflammatory infiltrates and rimmed vacuoles were visualised; typical 15‐ to 18‐nm filaments were documented for those patients for whom electron microscopy was available, protein accumulation evident by SMI‐31, TDP43‐ or p62‐positive aggregates was shown for the remaining. A brief overview of core clinical details of patients and controls is provided in Table S1.

### 2.2 Sample Preparation for iTRAQ Labelling and Mass Spectrometry Analysis

Samples for isobaric Tags for Relative and Absolute Quantification (iTRAQ) labelling and mass spectrometry were prepared as previously published [18]. Peptides from each lysed muscle biopsy sample were labelled using iTRAQ reagents 8plex (Sciex) according to the manufacturer's instructions and were separated on an offline 2D‐liquid chromatography (LC) system (Dionex, ULTIMATE 3000, Thermo Scientific), consisting of a 15‐cm strong cationic exchange column and a 25‐cm nano‐RP C18 column. The nano‐LC was coupled online to a QExactive‐Plus Orbitrap (Thermo Scientific) mass spectrometer (MS).

### 2.3 Bioinformatic Analysis of Mass Spectrometry Data

The generated raw MS data were processed with the Proteome Discoverer 2.1 (PD2.1) software (Thermo Scientific). For protein identification, Sequest HT was used against the human UniProt/SwissProt database with a false discovery rate (FDR) of less than 1%. The quantitative proteomics data were analysed with Perseus (Version 1.6.15.0) [19]. Abundance values generated by the PD2.1 software were Log_2_‐transformed and normalised by subtraction of the median value of the respective column. The complete list of identified proteins with the original scaled abundance values is available on request. Principal component analysis (PCA) and hierarchic clustering analyses using Euclidean algorithms were performed on 100% valid values, for hierarchic clustering after filtering ANOVA‐significantly dysregulated proteins (FDR = 0.01) and *Z*‐score transformation of values. Further analyses were based on proteins that were detected in at least 70% of the samples. A volcano plot analysis, assessing statistical significance (*t* test) together with fold change, was performed to identify significantly dysregulated proteins between patients and controls: A permutation‐based FDR cut‐off was determined with 250 randomisations and S0 = 0.1 (default). The downstream canonical pathways analysis (filtering based on the *p* value of overlap) and the upstream regulator analysis (filtering based on molecule type [genes, RNAs and proteins] and |*Z* score| ≥ 2 and *p* value of overlap ≤ 0.05) as a causal analysis approach were applied on this set of dysregulated proteins, using Ingenuity Pathway Analysis (IPA) (QIAGEN) [20].

### 2.4 IHC of Muscle Biopsy Samples

Frozen 7‐μm sections of skeletal muscle biopsies of four IBM patients and four healthy controls were mounted on Superfrost Plus glass slides (Thermo Fisher Scientific; 12‐550‐15). Additionally, patients from two other distinct IIM subtypes were included to explore the specificity of our findings in IBM: four dermatomyositis (DM) patients and four immune‐mediated necrotising myopathy (IMNM) patients. Core clinical details of patients included in these IHC experiments are provided in Table S2.

Sections were air‐dried and encircled using the ImmEdge pen to create a hydrophobic barrier. Muscle tissue sections were fixed in ice‐cold acetone for 10 min, washed with Tris‐buffered saline (TBS) three times for 5 min and incubated with blocking solution containing 4% goat serum and 1% BSA in TBS for 1 h at room temperature, followed by overnight incubation of rabbit‐anti‐KDM5A (1:50, Cell signal, #3876), mouse‐anti‐myogenin (1:50, Santa Cruz, sc‐12732), mouse‐anti‐CD68 (1:300, DAKO, M0718) and mouse‐anti‐CD8 (1:500, DAKO, M0707) primary antibodies at 4°C. After primary antibody incubation, sections were washed three times for 10 min with TBS and incubated with secondary antibodies, Alexa Fluor594‐conjugated goat‐anti‐rabbit (A11037, Life Technologies) and Alexa Fluor488‐conjugated goat‐anti‐mouse (A11001, Life Technologies), in blocking solution for 1 h at room temperature in the dark. Next, sections were washed twice with TBS for 5 min, quenched in a solution of 0.1% Sudan Black/70% ethanol for 10 min and washed with TBS for another 5 min. Nuclei were counterstained with Hoechst 33342 (1:20 000 dilution in TBS) for 10 min. Sections were then mounted with DAKO mounting medium (DAKO, S3023) onto microscope slides and stored at 4°C to allow the DAKO to set. Images were acquired with a Nikon Ti2 microscope with Celesta Crest X‐light V3 spinning disk, using a 40×/0.95 Plan Apochromat objective. Of each slide, three z‐stacks per tissue section were acquired, accumulating to nine z‐stacks per slide.

### 2.5 Human Myoblast Culture

Human myoblasts isolated from quadriceps muscle from a 53‐year‐old healthy male and immortalised as previously described were provided anonymously by MyoLine, from the Myology Institute in Paris [21]. Original sample was provided by Myobank, a tissue bank affiliated with EUROBIOBANK which is authorised by the French Ministry for Research to distribute anonymously human samples (authorisation AC‐2019‐3502). Human myoblasts were cultured in Skeletal Muscle Cell Medium (PromoCell; C‐23060) at 37°C in a humidified atmosphere containing 5% CO_2_. Myoblasts were differentiated to myotubes in DMEM + Glutamax high glucose (Life Sciences; 11574516) supplemented with 2% horse serum (Life Technologies; 26050070) and 1% penicillin–streptomycin (Life Technologies; 15140122) in Ibidi 96‐well plates (Ibidi; 89626) for immunostainings or on Geltrex‐coated tissue culture plates (Thermo Fisher; A1313301) for western blotting. To induce and mimic the IBM inflammatory environment, the differentiation medium was supplemented on Day 4 of differentiation for 72 h with 10‐ng/mL TNFα (purchased from the VIB‐UGent proteomics core), following established models [9]. At the same time point (Day 4), the differentiation medium was supplemented with 0.5‐μM ryuvidine (Bio‐Techne; 2609/10), a KDM5A‐specific inhibitor [22]. Cell cultures were fixated at Day 7 of differentiation with 4% paraformaldehyde in phosphate‐buffered saline (PBS), washed three times with PBS and stored at 4°C until staining.

### 2.6 Immunoblotting

Protein extractions of cell pellets were subjected to western blotting. Whole‐cell lysates were loaded and separated on 4%–12% NuPAGE Bis‐Tris gels (Life Technologies; 10247002) and transferred on Protran Premium 0.45‐μm nitrocellulose membranes (Amersham Biosciences; 10600003). Membranes were probed with rabbit‐anti‐KDM5A (1:1000 dilution, Thermo Fisher; MA5‐34682) and rabbit‐anti‐GAPDH as loading control (1:1000 dilution, GeneTex; GTX100118). Immunodetection was performed using host‐specific HRP‐conjugated secondary antibodies (goat‐anti‐rabbit, Jackson ImmunoResearch; 111‐035‐144), SuperSignal West Femto Maximum Sensitivity Substrate (Thermo Fisher Scientific; 34096) for KDM5A and Pierce ECL Plus Western Blotting Substrate (Thermo Fisher Scientific; 32132) for GAPDH. Western blots were visualised using the Amersham Imager 680 digital imaging system (GE Healthcare).

### 2.7 Immunocytochemistry

Differentiated myotubes were stained for p62 and APP. In brief, PBS was aspirated, and wells were blocked with normal donkey serum (Jackson ImmunoResearch; 017‐000‐001) 1:500 in PBT (PBS with 0.5% BSA and 0.5% Triton X‐100) for an hour at room temperature. Next, samples were incubated with either mouse‐anti‐p62 (Abcam; ab56416) or mouse‐anti‐APP (Fisher Scientific; 15577626) at 1:1000 dilution in PBT for 2 h at room temperature. Wells were washed three times with PBS, followed by incubation with Alexa Fluor647‐conjugated donkey‐anti‐mouse secondary antibody (Thermo Fisher; A21203) for an hour at room temperature. Nuclear staining was performed with Hoechst 33342 1:20 000 dilution (Life Technologies; H3570) in PBT for 10 min, followed by three washes with PBS. Image z‐stacks were acquired with a Nikon Ti2 microscope using a 20×/0.8 Plan Apochromat objective. Per well, eight random positions were automatically imaged using the JOBS module of Nikon NIS Elements software. Image z‐stacks were converted to maximum intensity projections for further image analysis with CellProfiler.

### 2.8 Image Analysis and Statistical Analysis

Immunoreactivity of KDM5A and cell type markers (myogenin, CD68 and CD8) in muscle tissue sections was analysed using the Fiji distribution of ImageJ [23]. In brief, image dimensions were rescaled to 1024 × 1024 pixels, and individual nuclei were segmented based on the nuclear Hoechst 33342 channel using the StarDist Fiji plugin and stored in the ImageJ ROI manager [24]. Mean intensities in nuclear ROIs were extracted from the KDM5A and cell marker fluorescence channels after background subtraction (rolling ball method with 200‐pixel radius). Intensities per nucleus were saved from ImageJ as .csv tables and further processed in Microsoft Excel. For each channel, a cut‐off intensity threshold was set to determine KDM5A‐positive nuclei and nuclei double positive for KDM5A and the cell marker. Further analysis was performed using GraphPad Prism 8.0.1 software for visualisation and statistical testing. Differences between percentages of marker‐positive nuclei (myogenin, CD8 and CD68) were tested with mixed‐model ANOVA with Tukey's post hoc to correct for multiple testing and intragroup variance between subjects. For KDM5A intensity, all segmented nuclei were pooled per group and tested with Mann–Whitney *U* test or Kruskal–Wallis with Dunn's post hoc to correct for multiple testing.

Maximum intensity projection images of three independent in vitro differentiation experiments were analysed using CellProfiler 4.2.5 (Cimini Lab, Broad Institute) to quantify the number of aggregates, cumulative aggregate area and the mean intensity of the p62 and APP signals in every image, as a measure for their cellular abundance [25]. The obtained values were pooled in Microsoft Excel and normalised to the negative control per experiment. Further analysis of these experiments was performed using GraphPad Prism 8.0.1 software for visualisation and statistical testing. Kruskal–Wallis with Dunn's post hoc test to correct for multiple testing was applied. *p* < 0.05 was considered as statistically significant for all tests.

## 3 Results

### 3.1 IBM Patients Demonstrate Distinct Proteomic Signatures in Skeletal Muscle

**FIGURE 1:**
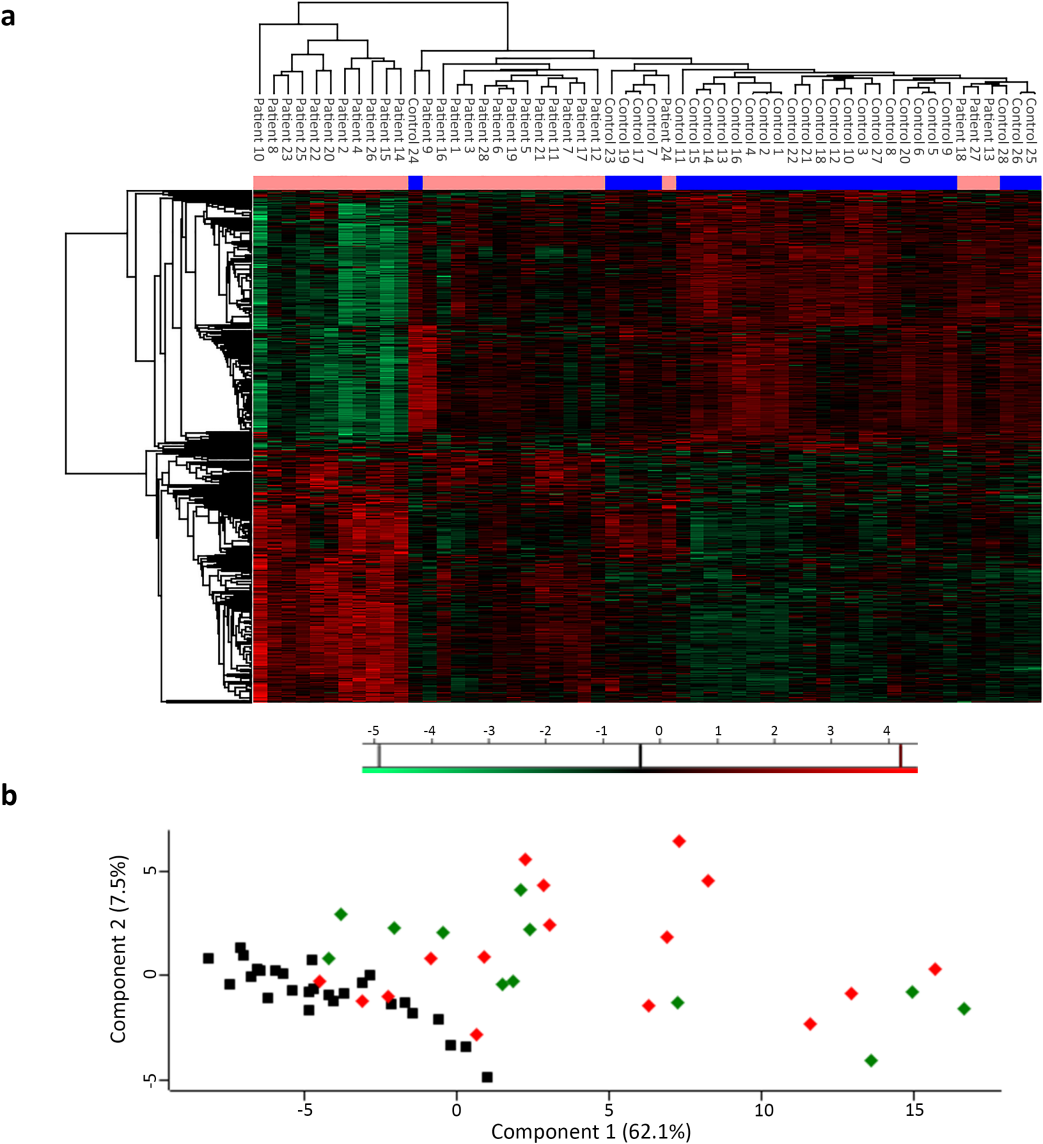
IBM patients cluster separately from controls based on muscle proteome profiles. (A) Hierarchical clustering analysis (with Euclidean algorithms) of the proteomic data of muscle tissue of 28 control individuals and 28 IBM patients. Colour codes of the intensities corresponding to the values that were normalised to the median across the complete dataset are shown below the heatmap. On top of the heatmap, patients are marked in pink, control individuals in blue. Twelve patients and control individuals are female (Patients 1–12 and Controls 1–12), 16 are male (Patients 13–28 and Controls 13–28). (B) Principal component analysis of protein expression data of controls (black squares), female (green diamonds) and male patients (red diamonds).

To identify a putative IBM disease‐related molecular signature in an unbiased way, we applied iTRAQ peptide labelling on skeletal muscle tissue lysates of 28 IBM patients and 28 matched controls to investigate the IBM proteomic landscape. In total, 3057 labelled peptides across all samples were detected, 1283 of which were common in at least 70% of the samples and 832 in all. Employing hierarchical clustering analysis and PCA on the proteomics, we found clear separation between IBM patient and control proteomes, with four patients clustering with controls (Figure 1A) in the hierarchical clustering, while in the PCA, based on Components 1 and 2 accounting for 62.1% and 7.5% of variance respectively, an additional female patient clustered with controls (Figure 1B).

**FIGURE 2:**
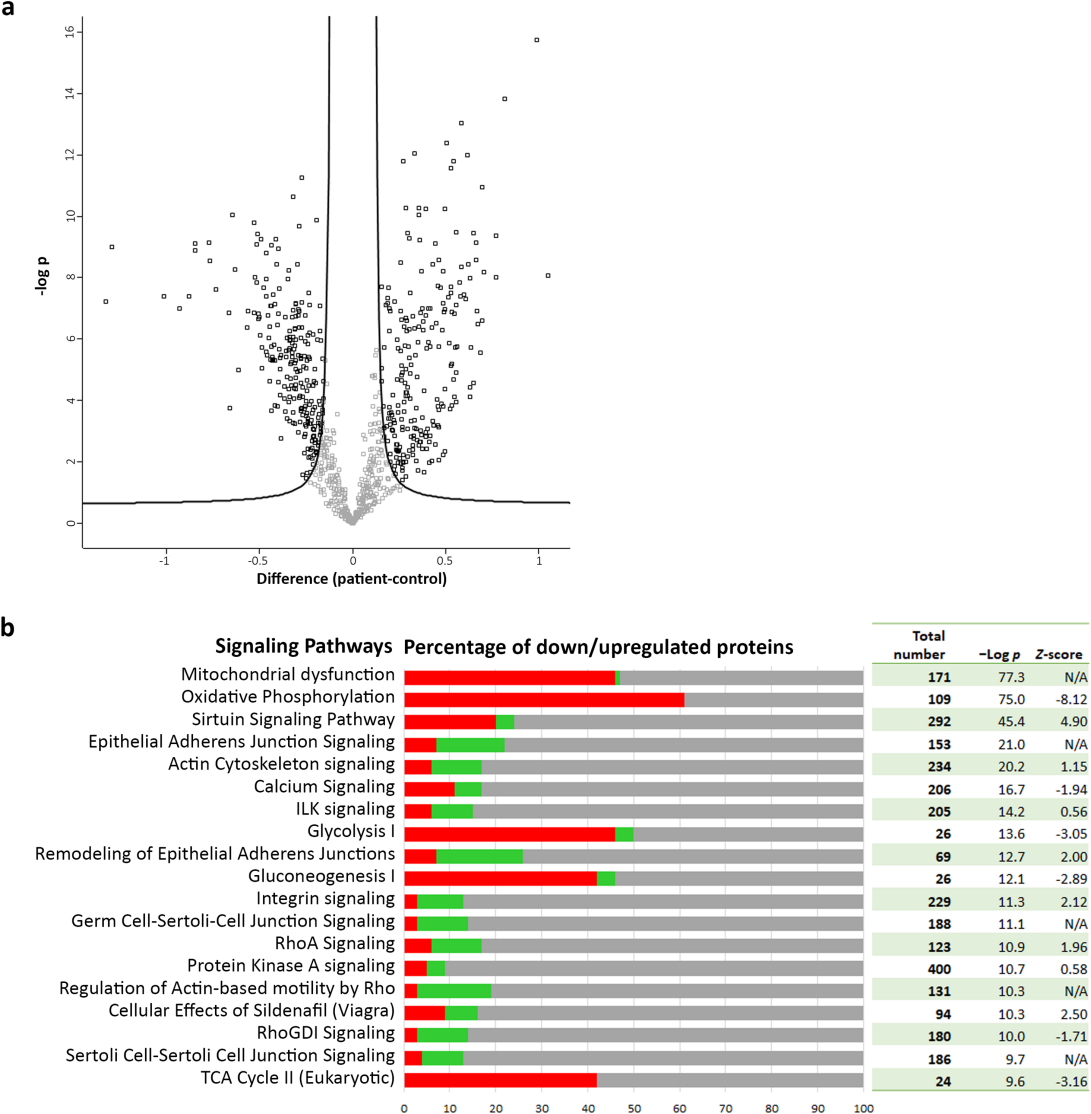
Volcano plot and downstream pathway analysis of the quantitative proteomic dataset. (A) Volcano plot analysis showing significantly dysregulated proteins (false discovery rate = 0.01, S0 = 0.1) between 28 control individuals and 28 IBM patients. The vertical axis corresponds to statistical significance (−Log *p*), the horizontal axis corresponds to the average fold change between patients and control individuals (difference in Log_2_ values). Squares marked in black represent significantly dysregulated proteins. (B) Results of the IPA canonical pathway analysis of significantly dysregulated proteins. Bar chart showing the proportion of downregulated (in red) and upregulated (in green) proteins as a percentage of the total number of molecules of the pathway as annotated in IPA. Filtering was based on *p* value of overlap. Pathways with a negative *Z* score are predicted to be inhibited, those with a positive *Z* score predicted to be activated. N/A, no activity pattern available.

Subsequently, to create a general appreciation of IBM disease signatures in patients' muscles, a volcano plot was generated to compare protein expression data of the 28 patients and matched controls, followed by functional annotation of significantly dysregulated proteins. Based on the volcano plot analysis (FDR = 0.01, S0 = 0.1), 627 proteins were significantly dysregulated, of which 313 were downregulated in patients (Figure 2A, full list of these proteins is provided in Table S3). Based on the *p* value of overlap, the top 20 enriched pathways identified in the canonical pathway analysis mainly reflected changes in metabolic pathways and cytoskeletal reorganisation (Figure 2B).

As we observed our patient cohort separating into two major subclusters, we assessed technical (e.g., variability between multiplexed sample runs) and biological factors including sex, muscle type, age at biopsy or disease duration but found no evident potential biases driving this subclustering. One subcluster of 11 patients exhibits, compared to their matched controls, significant alterations predominantly in mitochondrial protein expression and was found to display a proteomics signature similar (Table S4) to the signature of the whole cohort in both the downstream pathway (Figure 2B) and upstream regulator analyses (Table 1). The other subcluster of 17 patients demonstrates a primarily inflammatory signature (Table S4).

**TABLE 1:** Top upstream regulators as predicted by Ingenuity Pathway Analysis (IPA).

Upstream regulator	UniProt accession number	Molecule type	Activation *Z* score	*p* value of overlap
KDM5A	P29375	Transcription regulator	6.172	1.03E‐33
RICTOR	Q6R327	Other	5.511	1.01E‐39
OSM	P13725	Cytokine	5.204	4.53E‐10
MAP 4K4	O95819	Kinase	4.536	7.69E‐19
IL6	P05231	Cytokine	3.629	0.000091
NRG1	Q02297	Growth factor	3.616	1.63E‐08
IL4	P05112	Cytokine	3.612	1.64E‐10
SMTNL1	A8MU46	Other	3.606	1.72E‐13
TGFB1	P01137	Growth factor	3.59	1.57E‐21
GATA6	Q92908	Transcription regulator	3.259	0.00243
TSC2	P49815	Other	−3.363	4.91E‐08
MYC	P01106	Transcription regulator	−3.381	5.75E‐26
IGF1R	P08069	Transmembrane receptor	−3.431	4.73E‐21
PPARGC1B	Q86YN6	Transcription regulator	−3.632	5.21E‐13
ESRRA	P11474	Ligand‐dependent nuclear receptor	−3.766	2.59E‐08
MEF2C	Q06413	Transcription regulator	−4.021	1.78E‐15
PPARGC1A	Q9UBK2	Transcription regulator	−4.506	1.78E‐23
HBA1/HBA2	P69905	Transporter	−4.899	1.69E‐26
INSR	P06213	Kinase	−5.233	1.09E‐26
RB1	P06400	Transcription regulator	−5.964	2.49E‐17

*Note:* Top 10 of upstream regulators predicted by IPA to be activated or inhibited, based on proteins significantly dysregulated between 28 control individuals and 28 IBM patients. Filtering based on *p* value of overlap ≤ 0.05, sorting based on |*Z* score|.

### 3.2 Upstream Regulator Analysis Identifies Dysregulation of KDM5A and RB1 Axis as Candidate Master Regulators of Pathology

To investigate the proteins regulating upstream IBM pathology, we applied an upstream regulator analysis using IPA software to the significantly dysregulated proteins found across the whole patient cohort. This analysis identified proteins that were generally not directly detected with MS due to abundance below the detection limit, as is typically the case for key regulators. Within the top 10 activated and inhibited upstream regulators identified by IPA (Table 1, full list of predicted upstream regulators in Table S4), multiple regulators reflected: (1) inflammatory signatures of IBM pathology (predicted activation of OSM, IL6 and IL4), (2) changes in cellular energy homeostasis (predicted inhibition of PPARGC1A, PPARGC1B, IGF1R, INSR, ESRRA and TSC2 and activation of RICTOR) and (3) altered myogenesis (predicted activation of NRG1, MAP 4K4 and predicted inhibition of MEF2C and RB1), hinting at upstream metabolic dysfunction and dysregulation of myogenic differentiation in IBM [26, 27].

KDM5A and RB1, respectively the most activated and inhibited regulators identified in our upstream analysis, are known interaction partners and through their transcriptional regulation of cell cycle and senescence, differentiation and metabolic processes, interconnect with myogenic differentiation and cellular energy homeostasis, emphasising their importance and relevance as candidate master regulators of IBM pathology [28, 29, 30]. Sustained inactivation of RB1 in differentiating myoblasts results in expansion of satellite cells and immature myoblasts and deficient muscle fibre formation and maturation. KDM5A overactivity, or loss of regulation by RB1, leads to defective differentiation through negative regulation of mitochondrial and sarcomeric proteins, as we observe in our proteomics results as well (Figure S1) [28, 29, 31, 32].

### 3.3 KDM5A Expression in Human Skeletal Muscle and Immature Regenerating Muscle Fibres in IBM Muscle

Identified as our top upregulated predicted key regulator based on the upstream regulator analysis, we prioritised KDM5A as focus of experiments using human skeletal muscle and in vitro cell modelling to assess its potential as a novel key regulator of IBM pathomechanisms.

**FIGURE 3:**
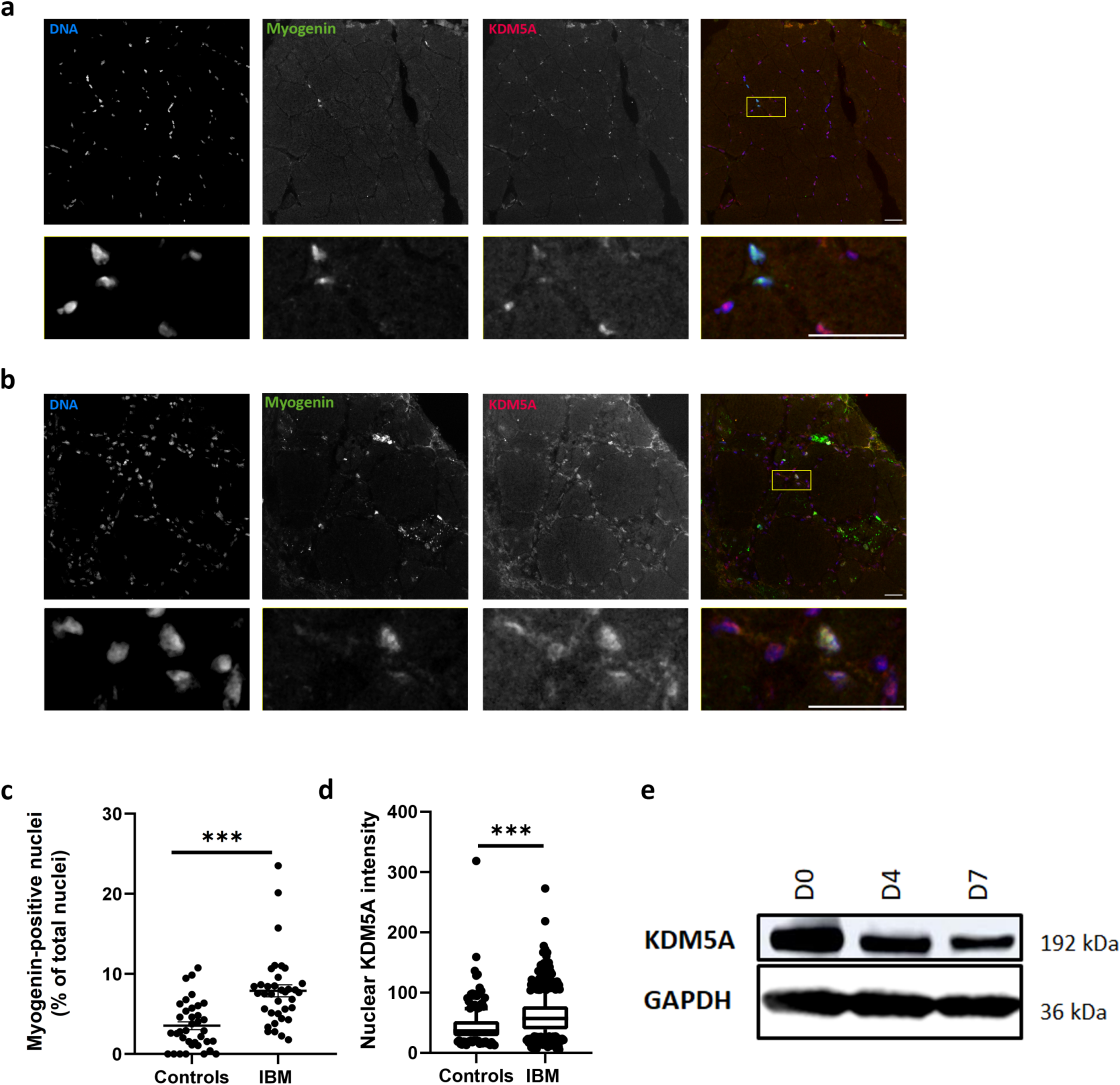
KDM5A colocalises with myogenin‐positive regenerating muscle fibres in IBM muscle and is expressed throughout myogenic differentiation, while lowly abundant in mature healthy myofibres. (A) Representative example of control and (B) IBM patient muscle tissue. Immunoreactivity for KDM5A is strictly nuclear in muscle tissue sections and predominantly localised to myogenin‐positive nuclei in regenerating muscle fibres of IBM patients. Scale bar overview and magnifications: 30 μm. (C) Quantification of percentage of myogenin‐positive myonuclei in healthy controls and IBM patients. Mean ± SEM; ***p < 0.0001. (D) Quantification of KDM5A intensity in myogenin‐positive myonuclei in healthy controls and IBM patients. Median, 10–90 percentile whiskers; ***p < 0.0001. (E) Immunoblotting of KDM5A in differentiating myoblasts in vitro shows KDM5A downregulation during differentiation. D0, Day 0; D4, Day 4; D7, Day 7 of differentiation.

Applying IHC to human muscle tissue, we observed strictly nuclear localisation of KDM5A, particularly in myogenin‐positive nuclei in immature muscle fibres in early stages of myogenic differentiation (Figure 3A,B). Comparing IBM patients' muscle tissue with healthy controls, we observed an increased percentage of myogenin‐positive myonuclei in IBM (*p* < 0.0001) that also showed increased KDM5A levels (*p* < 0.0001) (Figure 3C,D). Additionally, compared to two distinct IIM subtypes (DM and IMNM), KDM5A levels in myogenin‐positive myonuclei were significantly higher in IBM compared to DM (*p* = 0.0014) and IMNM (*p* < 0.0001). However, both IIM subtypes still showed significantly higher KDM5A levels compared to healthy controls as well (both *p* < 0.0001) (Figure S2). In IBM, CD8^+^ and CD68^+^ immune cell numbers were increased compared to controls and other IIM subtypes. Variable KDM5A levels in both immune cell types were observed in IBM and IIM subtypes without consistent trends (Figures S3 and S4).

### 3.4 KDM5A Is Downregulated During Myogenic Differentiation In Vitro

As KDM5A is associated with myogenic differentiation and colocalises with myogenin during in vivo differentiation, we investigated KDM5A expression during in vitro myogenic differentiation. To this end, we performed western blotting of KDM5A on differentiated human immortalised myoblasts from a healthy control individual with samples from three time points during differentiation. We observed pronounced KDM5A levels at the start of the myoblast differentiation (Day 0), which gradually decreased at later timepoints concurring with a commitment to differentiation and fusion of myoblasts to myotubes (Day 4), and maturation of myotubes towards the end of differentiation (Day 7) (Figure 3E).

### 3.5 Inhibition of KDM5A in an IBM‐Like Cell Model Reverts Pathological Features

To test the hypothesis that KDM5A may be an upstream regulator of IBM pathology, we next investigated if its inhibition would modulate hallmark pathological features (p62 and APP aggregation) in a previously established IBM‐mimicking cellular model. We incubated differentiating myoblasts with 10‐ng/mL TNFα and evaluated the extent of APP aggregation [9, 33]. To modulate these features, we implemented a proof‐of‐concept inhibition of KDM5A by administration of 0.5‐μM ryuvidine [22].

**FIGURE 4:**
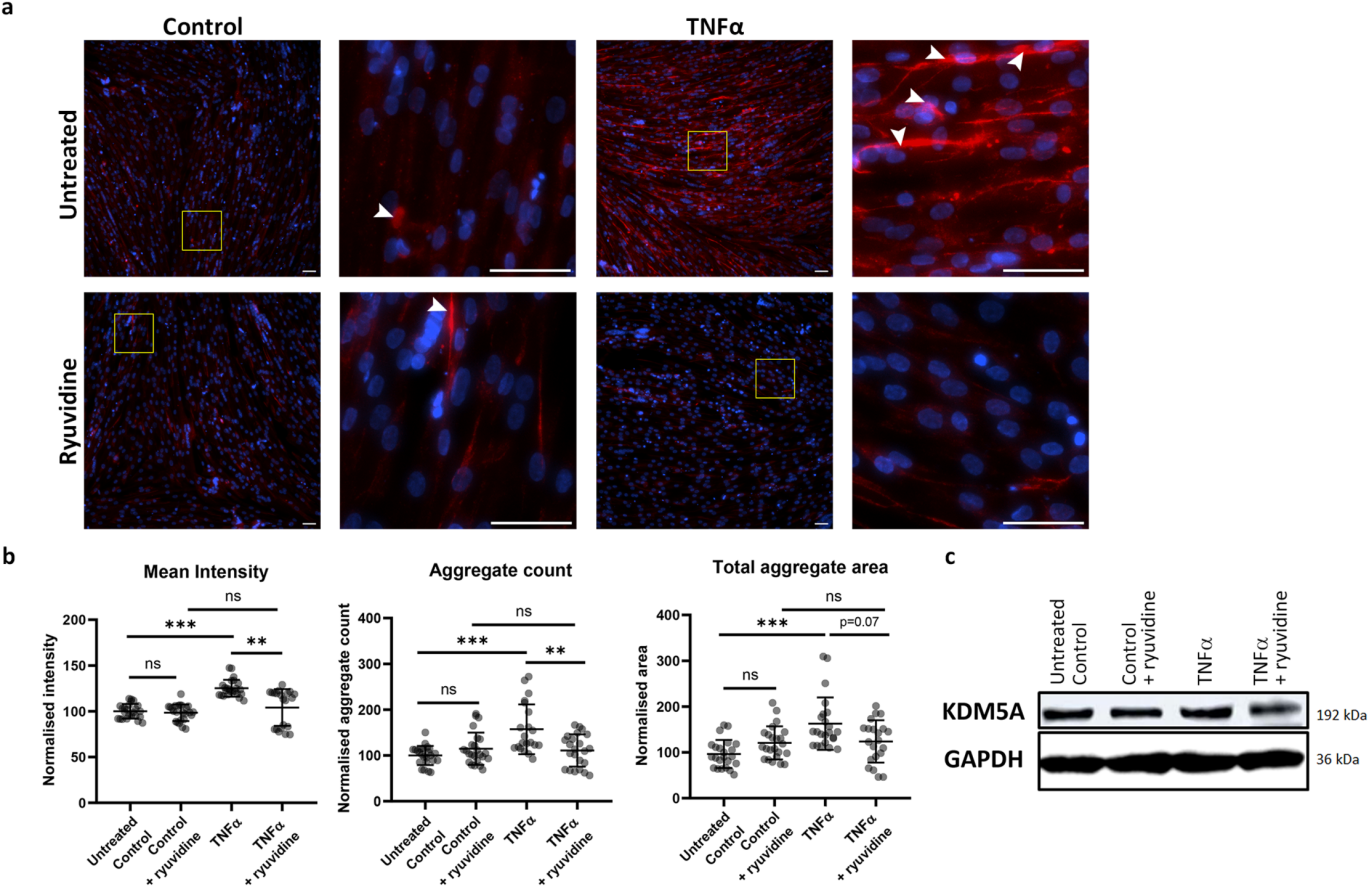
Inhibition of KDM5A in inflammatory IBM‐like cell model decreases APP abundance and aggregation. (A) Representative images and magnifications of APP immunostaining (red) and nuclear staining with Hoechst 33342 (blue) of differentiating human myoblasts at Day 7 upon treatment with KDM5A inhibitor ryuvidine and/or proinflammatory cytokine TNFα. White arrowheads indicate segmented APP aggregates. Scale bar overview and magnifications: 50 μm. (B) Quantification of APP accumulation by extracting the mean intensity, aggregate count and cumulative aggregate area in the myotubes. Pooled data from three independent biological replicate experiments and normalised to negative control to correct for interexperiment variation. Mean ± SD. ns, not significant; ***p < 0.0001; **p < 0.001; *p < 0.05. (C) Representative western blot probed for KDM5A and GAPDH as loading control.

Exposure to TNFα resulted in induction of pathological hallmarks, namely, significantly increased abundance (*p* = 0.001) and aggregation number (*p* < 0.0001) and total aggregate area (*p* < 0.0001) of APP compared to untreated myotubes (Figure 4A,B). APP abundance and aggregation levels were comparably low in ryuvidine‐treated and untreated control myotubes. Treating TNFα‐exposed myotubes with ryuvidine, we observed a statistically significant decrease compared to ryuvidine‐treated controls in both abundance (*p* = 0.0003) and aggregate count (*p* = 0.0132) (Figure 4B), whereas total APP aggregate area showed a decrease approaching statistical significance (*p* = 0.0733) upon inhibition of KDM5A.

In parallel, we investigated p62 abundance and aggregation properties upon KDM5A inhibition (Figure S5). TNFα treatment significantly increased both abundance and aggregation (all measured parameters *p* < 0.0001 compared to untreated controls). However, ryuvidine treatment did not induce significant changes in the measured parameters in our current experiments. Furthermore, we observed comparable KDM5A protein levels as determined by western blot in these different experimental conditions; thus, the ryuvidine treatment did not appear to affect KDM5A expression (Figure 4C).

Taken together, enzymatic inhibition of KDM5A with ryuvidine alleviated part of the disease hallmarks in the TNFα‐based cellular IBM model, providing a promising platform to further explore its potential involvement in reducing the cellular burden of APP and potentially alleviate the pressure on the dysfunctional ubiquitin–proteasome and autophagy pathways [11].

## 4 Discussion

In the present study, data from an unbiased proteomic approach shed unique insights into the proteomic landscape and disease signatures of IBM. Here, we report the largest proteomics dataset on IBM patient–derived muscle tissue published to date. Our data pointed to core cellular pathways previously associated with IBM pathomechanisms, suggesting that our approach was representative and valid. Leveraging this dataset, we identified KDM5A as a potential novel key disease regulator.

Previous proteomics studies in IBM were based on small patient numbers and focused on the downstream dysregulated proteins. While highly informative in terms of the downstream pathology, the crucial pivotal mechanisms early in the disease and upstream of pathology were not investigated [12, 15, 34]. As both inflammatory and degenerative features in IBM do not fully explain the disease aetiology and may well be downstream consequences, our study focused on novel upstream regulators of disease. Irrespective of any primary inflammatory or degenerative hypothesis, these upstream regulators may be hidden in the deep proteome, that is, lower abundant proteins regulating the function of detected proteins and their (differential) expression. Adding a layer of complexity to the analysis of the IBM proteomic dataset by conducting causal (upstream) analyses was therefore crucial to identify potential novel upstream regulators.

### 4.1 KDM5A Overactivity as Putative Upstream Factor in IBM Pathology

**FIGURE 5:**
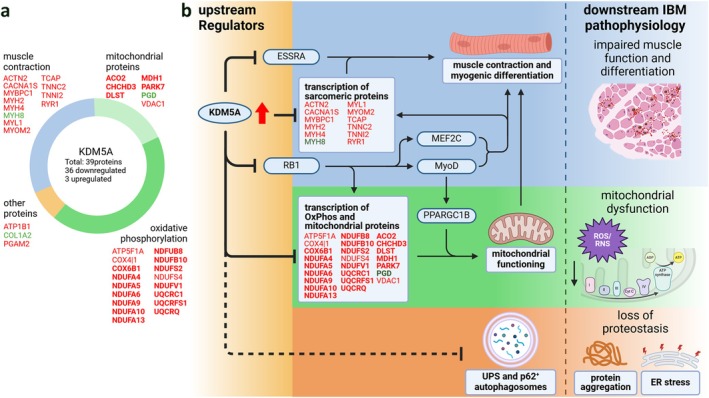
Graphical overview of the proteins downstream of KDM5A and the proposed mechanism by which KDM5A overactivity affects downstream processes associated with IBM pathology. (A) Diagram overview of KDM5A downstream targets that were identified with mass spectrometry and are predominantly downregulated by KDM5A in IBM muscle tissue. Proteins highlighted in bold were previously described as KDM5A targets by Váraljai et al. [29]. (B) Graphical representation of the proposed mechanism by which KDM5A is implicated in prominent features of IBM pathology via disruption of intermediate processes. Central to the proposed mechanism underlying (development of) IBM‐like pathology is the dysregulation of the KDM5A/RB1 axis, which regulates mitochondrial and myogenic functioning. KDM5A and RB1 regulate directly the expression of identified downstream proteins (listed in square boxes) that are differentially expressed in IBM patients compared to healthy controls (red: downregulated, green: upregulated; proteins marked in bold described as KDM5A targets by Váraljai et al.) and regulate the activity of several key upstream proteins (highlighted in elliptical boxes) identified in our IPA upstream regulator analysis, thereby impacting major downstream processes. Identified in our proteomics dataset as direct downstream targets of KDM5A, mitochondrial and sarcomeric proteins show altered expression in IBM. Their dysregulation is reflected in our downstream pathway analysis, which highlighted dysregulation of mitochondrial processes and oxidative phosphorylation, and alterations in mechanobiological processes. The dysregulation of sarcomeric proteins as consequence of overactivity of KDM5A and inhibition of RB1 results in changes in muscle contraction and function, thereby exacerbating the consequences of the inflammo‐degenerative environment on muscle fibres. Linking mitochondrial functioning to failure of myogenic differentiation as previously described, the dysregulated interaction between KDM5A and RB1 also impacts oxidative phosphorylation and metabolic homeostasis. The dysregulation of mitochondrial proteins in IBM leads to the increase in production of ROS/RNS and associated oxidative damage to proteins, rendering those prone to aggregation as is observed in IBM pathology. Finally, excessive protein aggregation can lead further downstream to ER stress and general loss of proteostasis. In our IBM‐like model, a hypothesised inhibitory effect of KDM5A overactivity on p62‐labelled autophagosomes remains currently unproven and is represented by a dashed inhibitory arrow. Potential links of KDM5A with other IBM‐like pathological features, such as ER stress, have not been investigated, though may be relevant in the context of KDM5A overactivity and loss of proteostasis [1, 8, 11, 29]. Created with biorender.com. IBM, inclusion body myositis; OxPhos, oxidative phosphorylation; ROS, reactive oxygen/nitrogen species; UPS, ubiquitin–proteasome system.

Our upstream regulator analysis revealed expected inflammatory changes and cellular energy metabolism disruptions, along with distinct signatures of dysfunctional myogenic differentiation as illustrated by the predicted activation of NRG1 and MAP 4K4 and inhibition of MEF2C and RB1 [26, 27, 31]. As KDM5A was previously shown to interlink mitochondrial dysfunction with failure of myogenic differentiation and several predicted key regulators and processes found in this study, it is clearly a candidate master upstream regulator of IBM pathological processes [29]. Although primary events leading to KDM5A dysregulation remain to be elucidated, KDM5A overactivity may trigger a cascade leading to failure of mitochondrial function, loss of proteostasis and failure of myogenic differentiation, which are all features of (pathological) muscle ageing (Figure 5) [35].

### 4.2 KDM5A and Muscle Regeneration in IBM Pathology

Our results place KDM5A at the heart of myogenic differentiation as we demonstrate increased KDM5A levels in myogenin‐positive regenerating muscle fibres in IBM compared to healthy controls and other IIM. KDM5A is physiologically present in myogenin‐positive myonuclei in healthy controls and downregulated upon differentiation (Figure 3E), underscoring its physiological role in conjunction with RB1 [30, 31]. The increased and persistent KDM5A levels in IBM may disrupt the physiological balance required for effective myogenic differentiation [29, 30]. This is in line with previously described results in non‐human cell lines, demonstrating that KDM5A overexpression has detrimental effects on proliferation of satellite cells, myoblast differentiation and myofibre formation and maturation [29]. Our data support the notion of an arrest of differentiation and maturation in IBM muscle as illustrated by the increased presence of myogenin‐positive myonuclei. This may also contribute to the premature ageing of the satellite cell niche and sarcopenia observed in IBM [36].

### 4.3 KDM5A and Ageing‐Associated Features in IBM Pathomechanisms

As age is currently the only factor strongly correlating with IBM, core features associated with ageing have yet to be studied systematically [11]. One ageing‐associated hallmark is DNA damage, typically induced by oxidative stress, and tends to accumulate with age [37]. Though KDM5A has a role in DNA double‐strand repair processes, it remains unknown if KDM5A overactivity contributes to accelerated ageing and, if so, through which mechanisms [29, 32, 38].

### 4.4 Exploration and Validation of Proteomics Findings In Vitro

The complex interplay of dysregulated processes in IBM (inflammation, degeneration and failure of differentiation) complicates the design of reliable experimental cell or animal models, emphasising the relevance of well‐designed experiments using human disease tissue. Prior IBM studies had already shown the strength of an unbiased proteomic approach, including identification of new risk alleles in IBM [18, 34]. Mass spectrometry–based technologies have evolved considerably [39]. Our findings allow for more simplified experimental design to study the dysfunction of processes in IBM in more detail through modulation, inhibition or (over)activation.

Although not strictly recapitulating the whole range of IBM pathological characteristics, the cell‐based model used in this study still has validity as IBM is increasingly recognised as a primarily IIM [6]. These in vitro experiments provided several additional insights into the direct biological role of KDM5A in IBM pathology. Our findings suggest that KDM5A overactivity contributes to IBM‐like pathology by influencing protein aggregation through still unknown intermediate processes, as demonstrated for APP as an aggregation marker. However, a direct link between KDM5A overactivity and dysfunction of autophagy remains unproven, as we observed no significant changes in p62‐positive aggregates following ryuvidine administration. A broader in vitro investigation into the relationship between KDM5A, autophagy and IBM‐like pathology was beyond the current scope. Nevertheless, our proof‐of‐concept findings suggest that KDM5A inhibition could be a potential therapeutic strategy to reverse degenerative features in IBM, an inflammo‐degenerative disease where inflammation, loss of proteostasis, metabolic homeostasis and myogenic differentiation are interconnected.

### 4.5 Study Limitations and General Challenges in IBM Research

Similar to every current study in IBM, our data can only predict the upstream drivers of IBM pathomechanisms. Our observations remain indirect, yet this dataset is exceedingly powered to allow an unbiased view of disease signatures as proximal to the aetiology as the current experimental setup allows. Studying the evolution of proteomic signatures (and disease progression) over time could yield additional valuable insights. However, stratifying patients based on disease stage is difficult (there is, e.g., no validated scoring system grading the severity of muscle biopsy changes) and obtaining serial invasive muscle biopsies is ethically difficult to justify.

Stratification based on known relevant clinical features did not explain the observed subclustering of our IBM patient cohort. Interestingly, this subclustering reflects the duality of IBM pathology with a predominantly inflammatory and mitochondrially driven subcluster respectively, the latter exhibiting patterns, similar to those found in the complete patient cohort. These observations might be indicative of more severe (degenerative) changes in IBM patients' muscle, potentially driven by KDM5A overactivity, and more specifically, what the sequence of events is leading up to KDM5A overactivity in IBM, that is, whether KDM5A is a true primary driver or triggered by a yet unknown stimulus. The subclustering may also simply reflect a more diverse proteomic profile and disease spectrum than previously assumed.

With our in vitro exploration of KDM5A as potential upstream regulator of IBM‐like pathology, we aimed to provide early proof‐of‐concept to supplement our proteomics findings in a low‐complexity in vitro model. Studies using more advanced in vitro models recapitulating the disease pathology closer may overcome this issue. In addition, further validation of these findings will need to be carried out in other described IBM‐like in vitro models based on exposure to IL‐1β, IFNγ or cytokine combinations or in other models based on, for example, KDM5A overexpression or IBM patient–derived myoblast cultures [9]. Lastly, the concentration of ryuvidine, the KDM5A inhibitor used in our experiments, might be insufficient to revert all IBM‐like in vitro pathological features. Other methods of KDM5A inhibition will need to be applied to study this further.

### 4.6 Future Outlooks

Due to the lack of effective therapy for IBM, the steady decline of muscle strength results in loss of ambulation and ultimately reduced life expectancy. As targeting both inflammatory and degenerative pathways in IBM with current treatments has proven to be unsuccessful, IBM research requires new therapeutic strategies based on novel and strong hypotheses and KDM5A might represent a relevant and druggable target [3, 4]. With inhibitors of KDM5 proteins being developed in cancer research, there might be opportunities for rapid trial readiness through drug repurposing [22, 40].

## 5 Conclusion

In summary, this unbiased proteomic study provides unique insights into the proteomic landscape of IBM, capturing known core features of IBM pathomechanisms and highlighting strong signatures pointing towards the selective failure of myogenesis. In addition, we propose that KDM5A overactivity is upstream of important epigenetic changes in mature muscle and immature myoblasts, leading to ageing‐associated skeletal muscle dysfunction and primary failure of muscle regeneration in IBM, in turn resulting in highly interconnected degenerative and inflammatory changes in skeletal muscle. These results position KDM5A overactivity and its downstream effects in IBM pathology as clear subjects of future studies.

## Ethics Statement

The authors certify they comply with the ethical guidelines for authorship and publishing. Ethical approval was granted by the relevant local Ethical Committees of the participating centres. All participants provided informed consent prior to participation in the study.

## Conflicts of Interest

J.L.D.B. is PI in an ongoing ABCURO study in sIBM patients. All other authors report no disclosures relevant to the manuscript.

## Supporting information


**Table S1.** Summarised clinical details of IBM patients and matched controls included in the study.
**Table S2.** Summarised clinical details of patients included in the immunohistochemical studies.
**Figure S1.** Volcano plot with KDM5A‐associated downstream targets differentially expressed in IBM highlighted.
**Figure S2.** Myogenin^+^ myonuclei and KDM5A intensity in healthy controls, IBM and IIM patients.
**Figure S3.** KDM5A presence in nuclei of CD8^+^ T‐cells in healthy controls, IBM and IIM patients.
**Figure S4.** KDM5A presence in nuclei of CD68^+^ macrophages in healthy controls, IBM and IIM patients.
**Figure S5.** Inhibition in IBM‐like cell model does not alter p62 aggregation or abundance under inflammatory conditions.

**Table S3.** Full list of dysregulated proteins based on volcano plot analysis.

**Table S4.** Full lists of proteins identified through upstream regulator analysis using Ingenuity Pathway Analysis software of our whole IBM patient cohort and the two separate subclusters against their matched controls.

## Data Availability

Anonymised data not published within the article will be shared upon request by qualified investigators.
