# Supplementary material for: Ageing Signatures and Disturbed Muscle Regeneration in Muscle Proteome of Inclusion Body Myositis
Source: J Cachexia Sarcopenia Muscle. 2025 Jun 8;16(3):e13845. doi: 10.1002/jcsm.13845 (PMC12146582; doi:10.1002/jcsm.13845)
Supplement: Supplementary file 1 — Table S1. Summarised clinical details of IBM patients and matched controls included in the study. Table S2. Summarised clinical details of patients included in the immunohistochemical studies. Figure S1. Volcano plot with KDM5A‐associated downstream targets differentially expressed in IBM highlighted. Figure S2. Myogenin+ myonuclei and KDM5A intensity in healthy controls, IBM and IIM patients. Figure S3. KDM5A presence in nuclei of CD8+ T‐cells in healthy controls, IBM and IIM patients. Figure S4. KDM5A presence in nuclei of CD68+ macrophages in healthy controls, IBM and IIM patients. Figure S5. Inhibition in IBM‐like cell model does not alter p62 aggregation or abundance under inflammatory conditions. [file JCSM-16-e13845-s003.docx]

**Ageing signatures and disturbed muscle regeneration in muscle proteome of inclusion body myositis**

Geert M. de Vries, MSc^1,2^; Bob Asselbergh, PhD^3,4^;Alice Monticelli, MSc^1,2^;Peter De Jonghe MD, PhD^2,5^;Stuart Maudsley, PhD^6^; Peter Y.K. Van Den Bergh MD, PhD^7^;Anne Bigot, PhD^8^; Jan L. De Bleecker, MD, PhD^9^; Biljana Ermanoska, PhD^1,2^;Willem De Ridder, MD, PhD*^1,2,5^; Jonathan Baets, MD, PhD*^1,2,5^

**Supplementary Material: content**

**Supplementary Table S1**

**Supplementary Table S2**

**Supplementary Table S3**

**Supplementary Table S4**

**Supplementary Figure S1**

**Supplementary Figure S2**

**Supplementary Figure S3**

**Supplementary Figure S4**

**Supplementary Figure S5**

**Supplementary Table S1. Summarised clinical details of IBM patients and matched controls included in the study**

| **Subject** | **Sex** | **Age at biopsy, y** | **Biopsied muscle** | **Approximate disease duration, y** | **Age at onset > 45y** | **Duration > 12m** | **Rimmed vacuoles** | **Endomysial infiltration** | **Mitochondrial abnormalities** |
| --- | --- | --- | --- | --- | --- | --- | --- | --- | --- |
| Patient 1 | Female | 57 | quadriceps | 1 | + | + | + | + | + |
| Patient 2 | Female | 65 | quadriceps | 10 | + | + | + | + | + |
| Patient 3 | Female | 70 | quadriceps | 8 | + | + | + | + | - |
| Patient 4 | Female | 75 | quadriceps | 4 | + | + | + | + | - |
| Patient 5 | Female | 62 | quadriceps | 5 | + | + | + | + | - |
| Patient 6 | Female | 63 | quadriceps | 1 | + | + | + | + | + |
| Patient 7 | Female | 70 | quadriceps | 1 | + | + | + | + | + |
| Patient 8 | Female | 85 | quadriceps | 2 | + | + | + | + | + |
| Patient 9 | Female | 55 | tibialis anterior | 3 | + | + | + | + | + |
| Patient 10 | Female | 68 | tibialis anterior | 18 | + | + | + | + | - |
| Patient 11 | Female | 78 | tibialis anterior | 4 | + | + | + | + | + |
| Patient 12 | Female | 83 | deltoid | 10 | + | + | + | + | + |
| Patient 13 | Male | 69 | quadriceps | 4 | + | + | + | + | + |
| Patient 14 | Male | 75 | quadriceps | 2 | + | + | + | + | + |
| Patient 15 | Male | 77 | quadriceps | 3 | + | + | + | + | + |
| Patient 16 | Male | 79 | quadriceps | 2 | + | + | + | + | + |
| Patient 17 | Male | 58 | quadriceps | 5 | + | + | + | + | - |
| Patient 18 | Male | 70 | quadriceps | 5 | + | + | + | + | - |
| Patient 19 | Male | 70 | quadriceps | 2 | + | + | + | + | + |
| Patient 20 | Male | 75 | quadriceps | 3 | + | + | + | + | + |
| Patient 21 | Male | 57 | tibialis anterior | 4 | + | + | + | + | + |
| Patient 22 | Male | 61 | tibialis anterior | Unknown, >12 months | + | + | + | + | + |
| Patient 23 | Male | 66 | tibialis anterior | 8 | + | + | + | + | + |
| Patient 24 | Male | 75 | tibialis anterior | 4 | + | + | + | + | - |
| Patient 25 | Male | 58 | quadriceps | Unknown, >12 months | + | + | + | + | + |
| Patient 26 | Male | 64 | quadriceps | 1 | + | + | + | + | + |
| Patient 27 | Male | 82 | quadriceps | 7 | + | + | + | + | + |
| Patient 28 | Male | 66 | deltoid | 3 | + | + | + | + | + |

| **Subject** | **Sex** | **Age at biopsy, y** | **Biopsied muscle** |
| --- | --- | --- | --- |
| Control 1 | Female | 50 | quadriceps |
| Control 2 | Female | 51 | quadriceps |
| Control 3 | Female | 66 | quadriceps |
| Control 4 | Female | 76 | quadriceps |
| Control 5 | Female | 52 | quadriceps |
| Control 6 | Female | 53 | quadriceps |
| Control 7 | Female | 68 | quadriceps |
| Control 8 | Female | 85 | quadriceps |
| Control 9 | Female | 58 | quadriceps |
| Control 10 | Female | 60 | quadriceps |
| Control 11 | Female | 64 | deltoid |
| Control 12 | Female | 76 | tibialis anterior |
| Control 13 | Male | 53 | quadriceps |
| Control 14 | Male | 59 | quadriceps |
| Control 15 | Male | 68 | quadriceps |
| Control 16 | Male | 88 | quadriceps |
| Control 17 | Male | 51 | quadriceps |
| Control 18 | Male | 59 | quadriceps |
| Control 19 | Male | 60 | quadriceps |
| Control 20 | Male | 73 | quadriceps |
| Control 21 | Male | 49 | tibialis anterior |
| Control 22 | Male | 56 | tibialis anterior |
| Control 23 | Male | 62 | tibialis anterior |
| Control 24 | Male | 65 | tibialis anterior |
| Control 25 | Male | 53 | quadriceps |
| Control 26 | Male | 58 | quadriceps |
| Control 27 | Male | 79 | quadriceps |
| Control 28 | Male | 76 | deltoid |

Overview of core clinical details and characteristics of patients and controls included in this study. IBM, inclusion body myositis; y, years, m, months.

**Supplementary Table S2. Summarised clinical details of patients included in the immunohistochemical studies**

| **Subject** | **Sex** | **Age at muscle biopsy, y** | **Biopsied muscle** |
| --- | --- | --- | --- |
| DM1 (MDA5) | Male | 76 | quadriceps |
| DM2 (TIF1γ) | Female | 63 | deltoid |
| DM3 (MDA5) | Female | 43 | quadriceps |
| DM4 (TIF1γ) | Male | 70 | deltoid |
|  |  |  |  |
| IMNM1 | Male | 60 | quadriceps |
| IMNM2 | Male | 66 | quadriceps |
| IMNM3 | Male | 86 | quadriceps |
| IMNM4 | Female | 76 | quadriceps |
|  |  |  |  |
| IBM1 | Male | 75 | quadriceps |
| IBM2 | Male | 57 | tibialis anterior |
| IBM3 | Male | 61 | tibialis anterior |
| IBM4 | Male | 66 | tibialis anterior |
|  |  |  |  |
| Control1 | Male | 88 | quadriceps |
| Control2 | Male | 51 | quadriceps |
| Control3 | Male | 65 | tibialis anterior |
| Control4 | Male | 68 | deltoid |

Overview of core clinical details and characteristics of patients and controls included in the immunohistochemistry experiment. y, years; DM, dermatomyositis; MDA5, melanoma differentiation-associated protein 5; TIF1γ, transcription intermediary factor 1 gamma; IMNM, immune-mediated necrotising myositis; IBM, inclusion body myositis.

**Supplementary Table S3**

Full list of dysregulated proteins based on volcano plot analysis. N.D.: not detected

Provided in a separate excel document for improved readability.

**Supplementary Table S4**

Full lists of proteins identified through upstream regulator analysis using Ingenuity Pathway Analysis software of our whole IBM patient cohort and the two separate subclusters against their matched controls.

For improved readability, data is provided in a separate Excel document with distinct tabs for each analysis:

| **Sheet name** | **Description** | **Important notes** |
| --- | --- | --- |
| Full_cohort | List of identified upstream regulators with IPA on the whole IBM patient cohort compared to their matched controls | Discussed under section “Upstream regulator analysis identifies dysregulation of KDM5A and RB1 axis as candidate master regulators of pathology”, lines 306-329 |
| Subcluster1_11pat | List of identified upstream regulators with IPA on a subcluster of 11 IBM patients compared to their matched controls | Referred to in main text as “one subcluster of 11 patients (…)”, lines 300-303. |
| Subcluster2_17pat | List of identified upstream regulators with IPA on a subcluster of 17 IBM patients compared to their matched controls | Referred to in main text as “The other subcluster of 17 patients (…)”, line 304. |


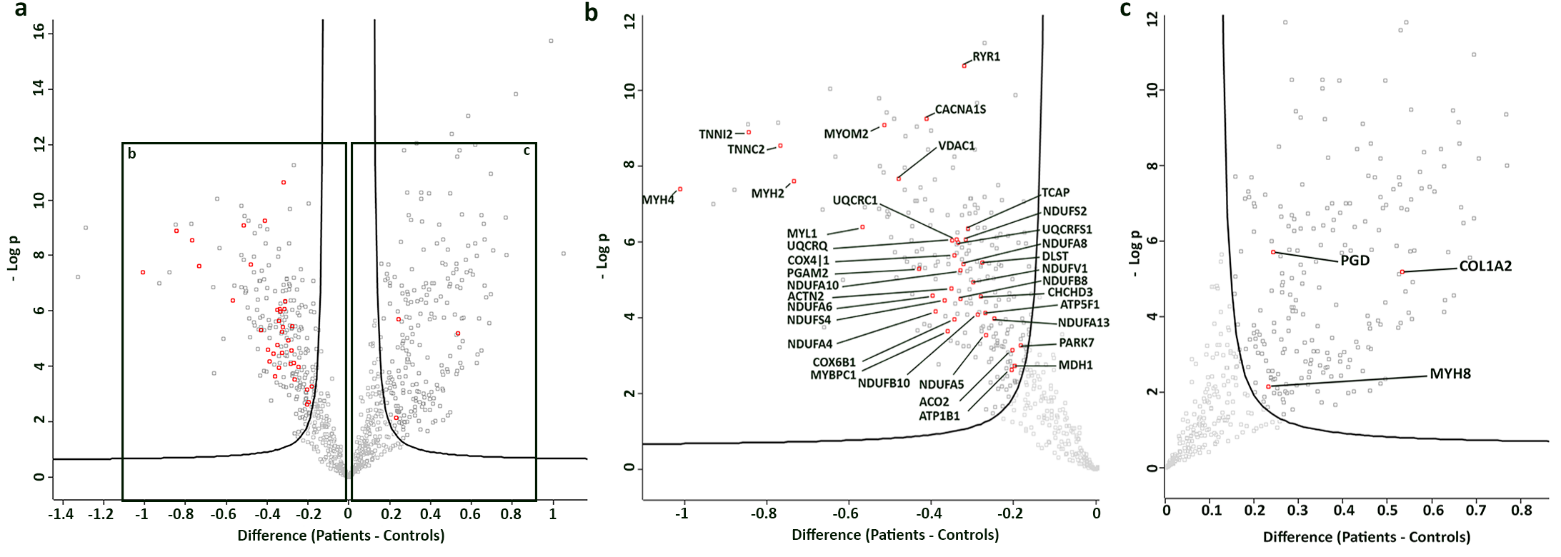


**Supplementary Figure S1. Volcano plot with KDM5A-associated downstream targets differentially expressed in IBM highlighted**

(A) Full volcano plot with KDM5A-associated downstream targets highlighted (red) and zoom boxes for panels B and C; (B) In IBM patients downregulated KDM5A-associated downstream targets highlighted and labelled. These proteins consist mostly of mitochondrial and sarcomeric proteins downregulated in IBM as consequence of KDM5A overactivity; (C) In IBM upregulated KDM5A-associated downstream targets highlighted and labelled.

**
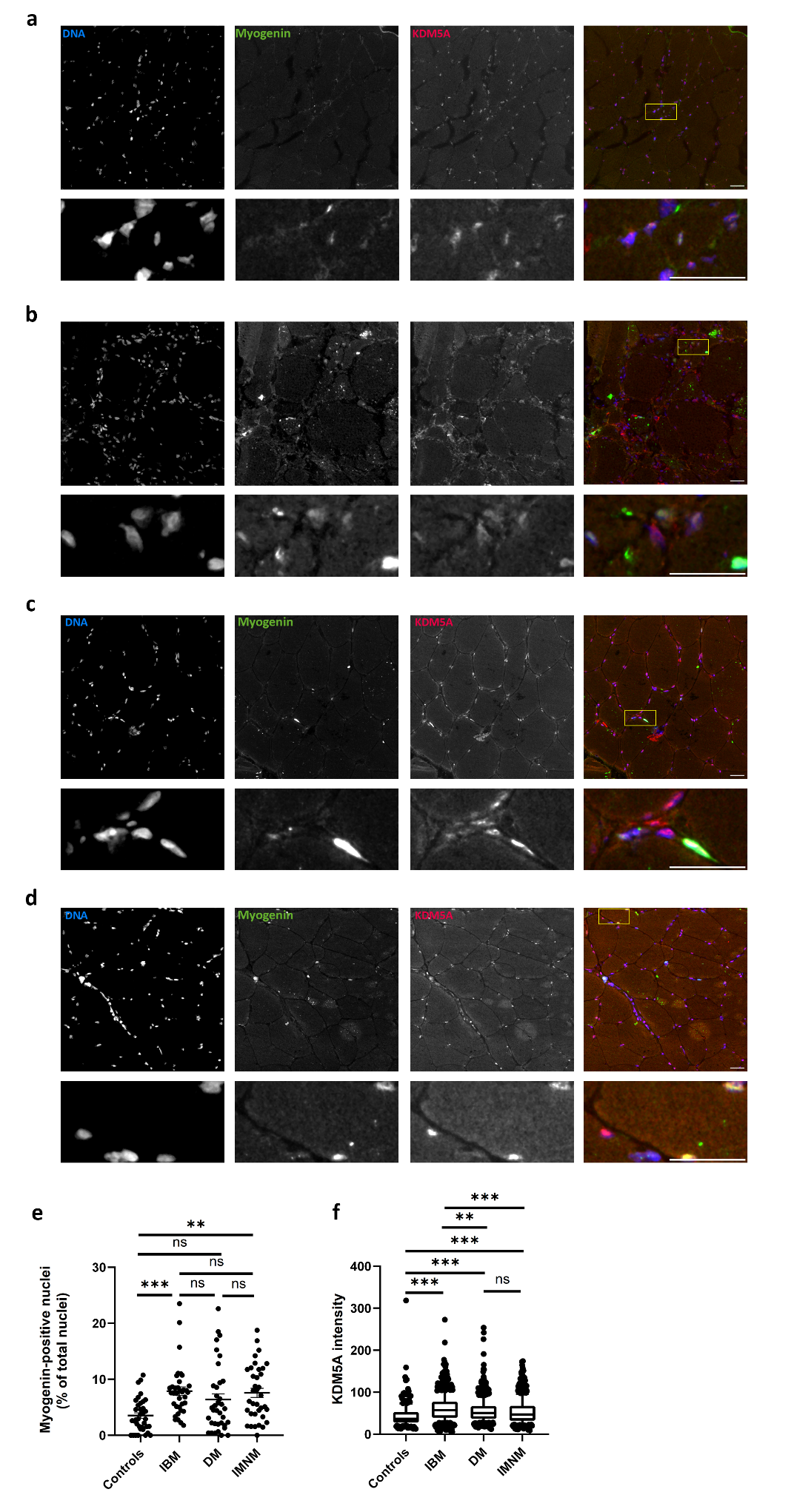
**

**Supplementary Figure S2. Myogenin^+^ myonuclei and KDM5A intensity in healthy controls, IBM and IIM patients**

(A) Myogenin^+^ myonuclei and KDM5A co-staining in muscles of healthy controls, (B) IBM patients, (C) DM patients, and (D) IMNM patients. Scale bar overview and magnifications: 30 μm. (E) Percentage of myogenin^+^ myonuclei as proportion of total nuclei in each image in each patient group. Data points represent individual images. Mean±SEM; (F) Nuclear KDM5A intensity in each patient group. Data points represent individual segmented myogenin-positive myonuclei. Median, 10-90^th^ percentile; *** p<0.0001; ** p<0.01; ns, not significant; IBM, inclusion-body myositis; IIM, idiopathic inflammatory myopathy; DM, dermatomyositis; IMNM, immune-mediated necrotising myositis.


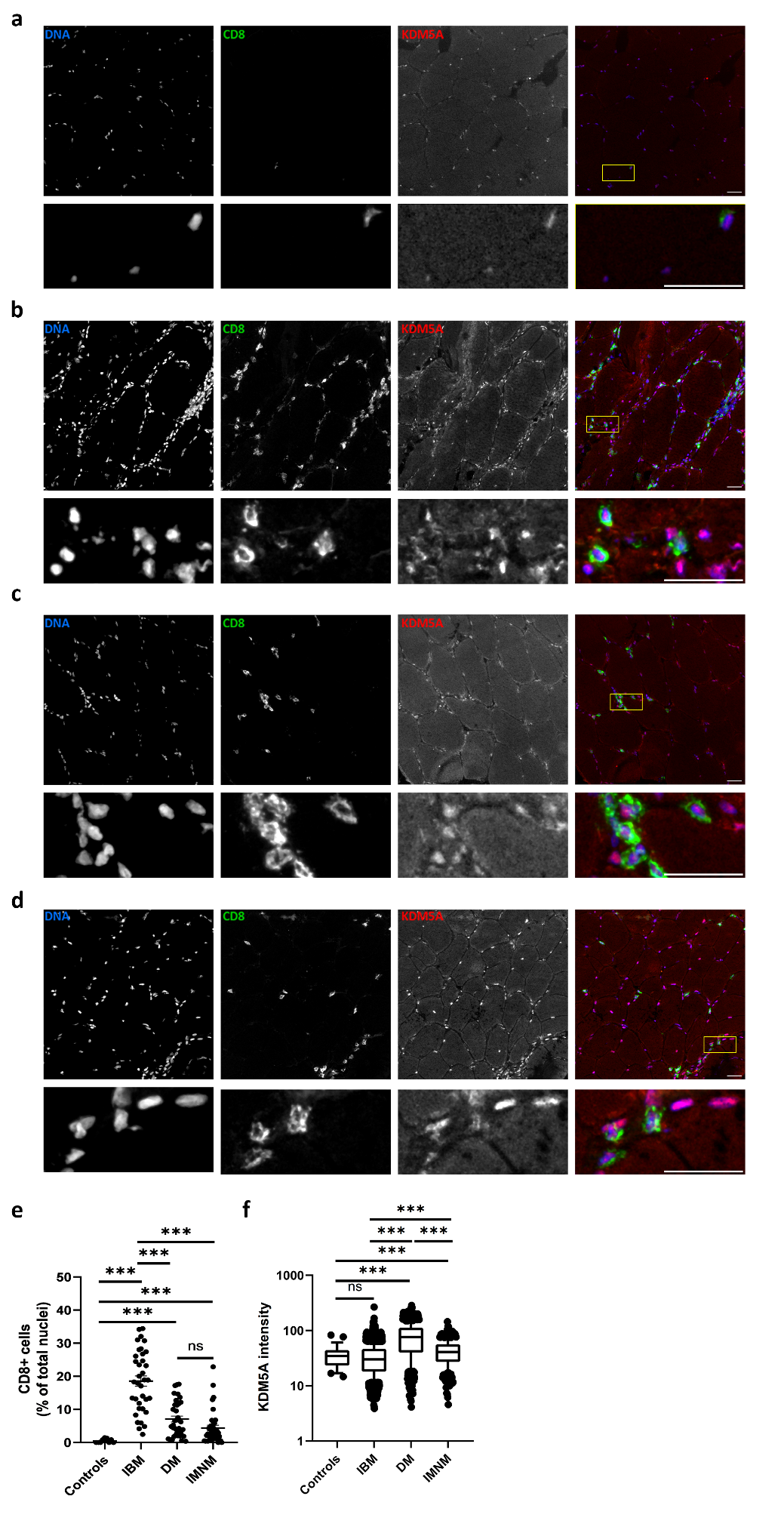


**C**

**Supplementary Figure S3. KDM5A presence in nuclei of CD8^+^ T-cells in healthy controls, IBM and IIM patients**

(A) KDM5A abundance in CD8^+^ infiltrating T-cells in muscles of healthy controls, (B) IBM patients, (C) DM patients, and (D) IMNM patients. Scale bar overview and magnifications: 30 μm (E) Percentage of CD8^+^ T-cells as proportion of total nuclei in each image in each patient group, demonstrating significantly higher numbers of CD8^+^ T-cells present in IBM compared to controls and other IIM subtypes. Data points represent individual images. Mean±SEM; (F) Nuclear KDM5A intensity in CD8^+^ T-cells in each patient group, log_10_ scale, showing no difference in KDM5A intensity in IBM compared to controls, and significantly lower levels in IBM compared other IIM subtypes. Data points represent individual segmented nuclei. Median, 10-90^th^ percentile; *** p<0.0001 ** p<0.01; ns, not significant; IBM, inclusion-body myositis; IIM, idiopathic inflammatory myopathy; DM, dermatomyositis; IMNM, immune-mediated necrotising myositis.

**
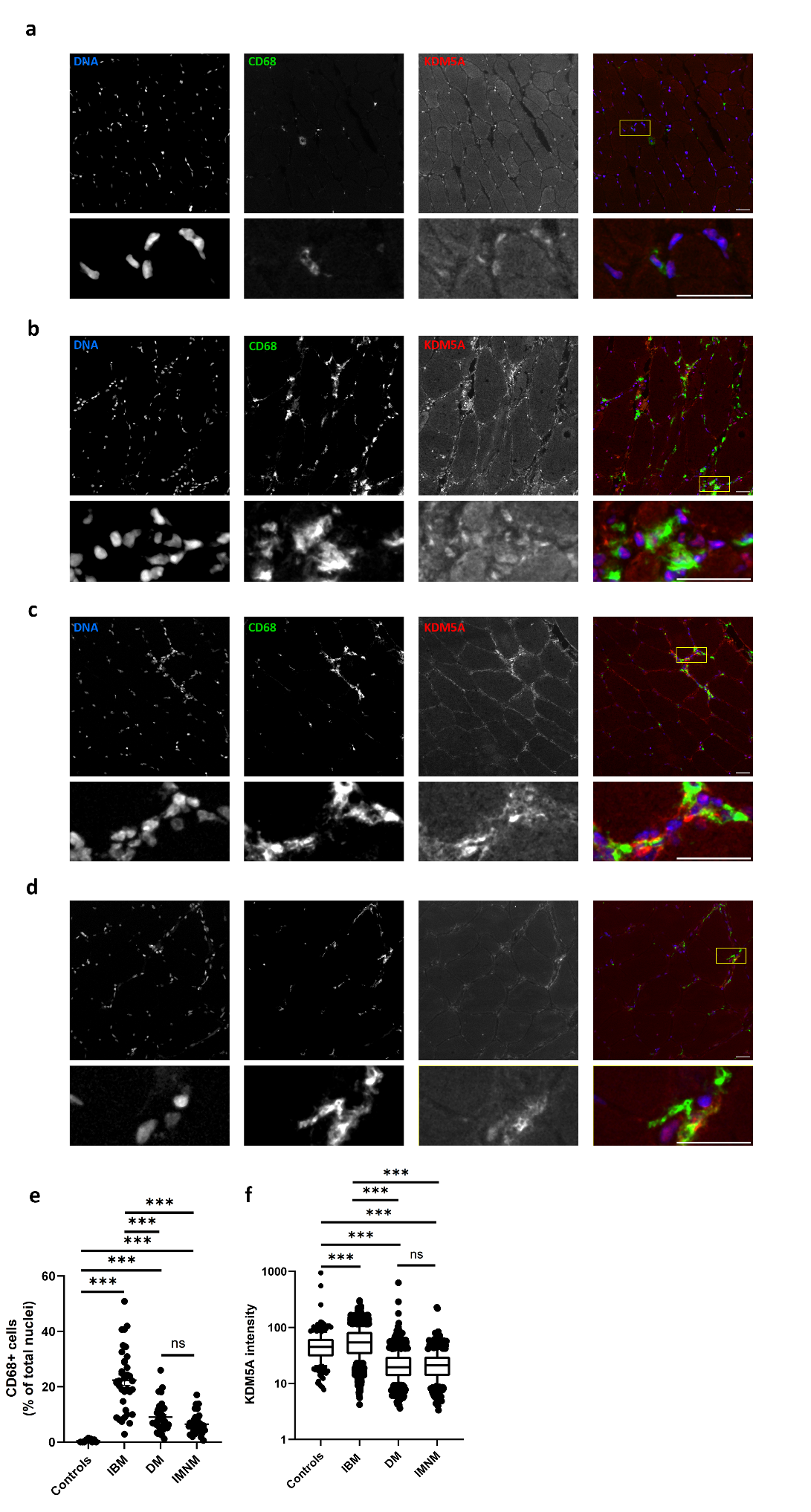
**

**Supplementary Figure S4. KDM5A presence in nuclei of CD68^+^ macrophages in healthy controls, IBM and IIM patients**

A) KDM5A abundance in CD68^+^ infiltrating macrophages in muscles of healthy controls, (B) IBM patients, (C) DM patients, and (D) IMNM patients. Scale bar overview and magnifications: 30 μm (E) Percentage of CD68^+^ macrophages as proportion of total nuclei in each image in each patient group, demonstrating significantly higher numbers of CD68^+^ macrophages present in IBM compared to controls and IIM subtypes. Data points represent individual images. Mean±SEM; (F) Nuclear KDM5A intensity in CD68^+^ macrophages each patient group, log_10_ scale, showing significantly higher KDM5A levels in CD68^+^ macrophages in IBM compared to controls and IIM subtypes. Data points represent individual segmented nuclei. Median, 10-90^th^ percentile; *** p<0.0001 ** p<0.01; ns, not significant; IBM, inclusion-body myositis; IIM, idiopathic inflammatory myopathy; DM, dermatomyositis; IMNM, immune-mediated necrotising myositis.

**
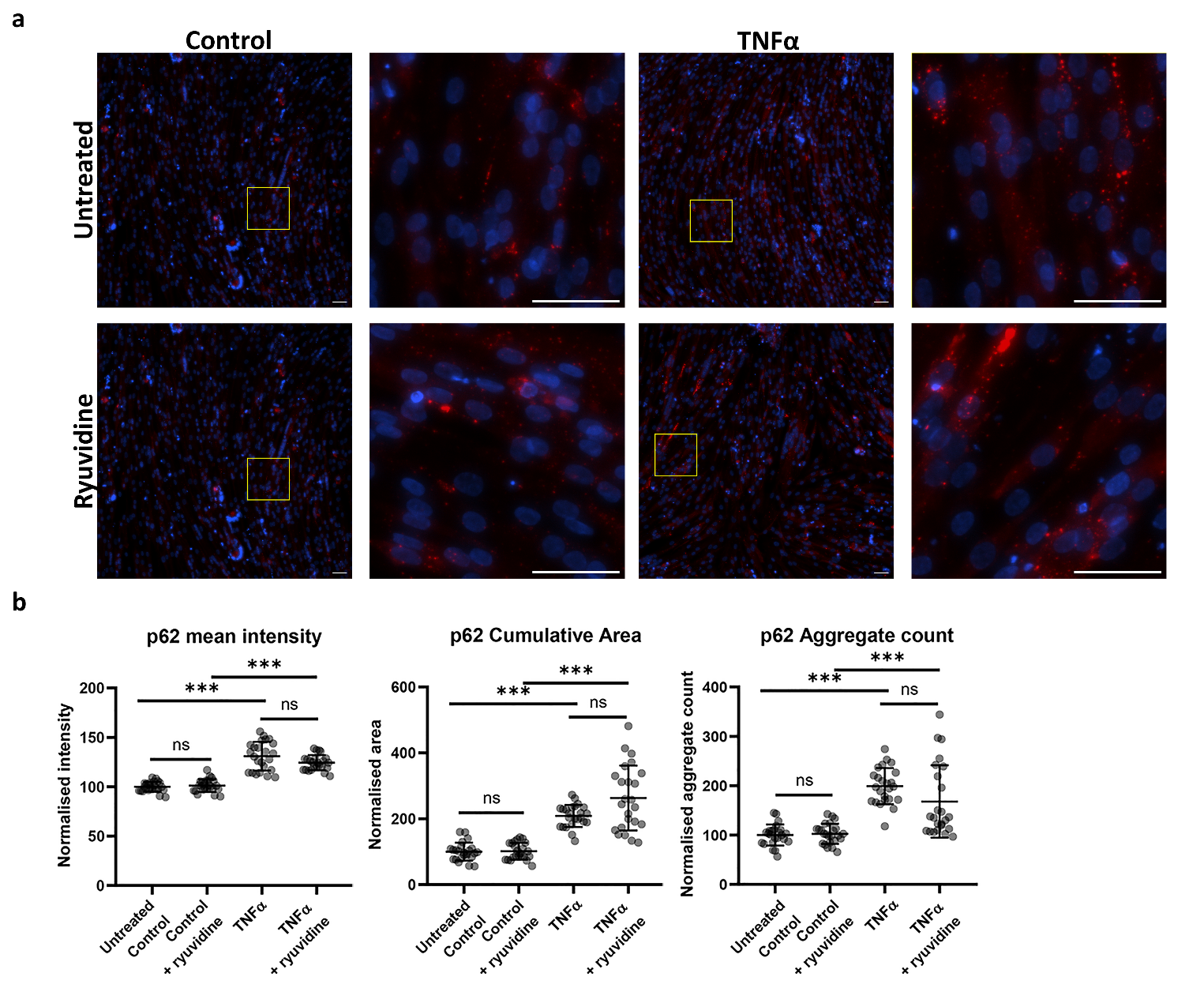
**

**Supplementary Figure S5. Inhibition in IBM-like cell model does not alter p62 aggregation or abundance under inflammatory conditions**

(A) representative images and magnifications of p62 immunostaining (red) and nuclear staining with Hoechst 33342 (blue) of differentiating human myoblasts at day 7 upon treatment with KDM5A inhibitor ryuvidine and/or proinflammatory cytokine TNFα. Scale bar overview and magnifications: 50 μm

(B) Quantification of p62 accumulation by extracting the mean intensity, aggregate count, and cumulative aggregate area in the myotubes. Pooled data from three independent biological replicate experiments and normalized to negative control to correct for inter-experiment variation. Mean±SD. Neg. ctrl., negative control; ctrl, control; ns, not significant; *** p<0.0001.

**
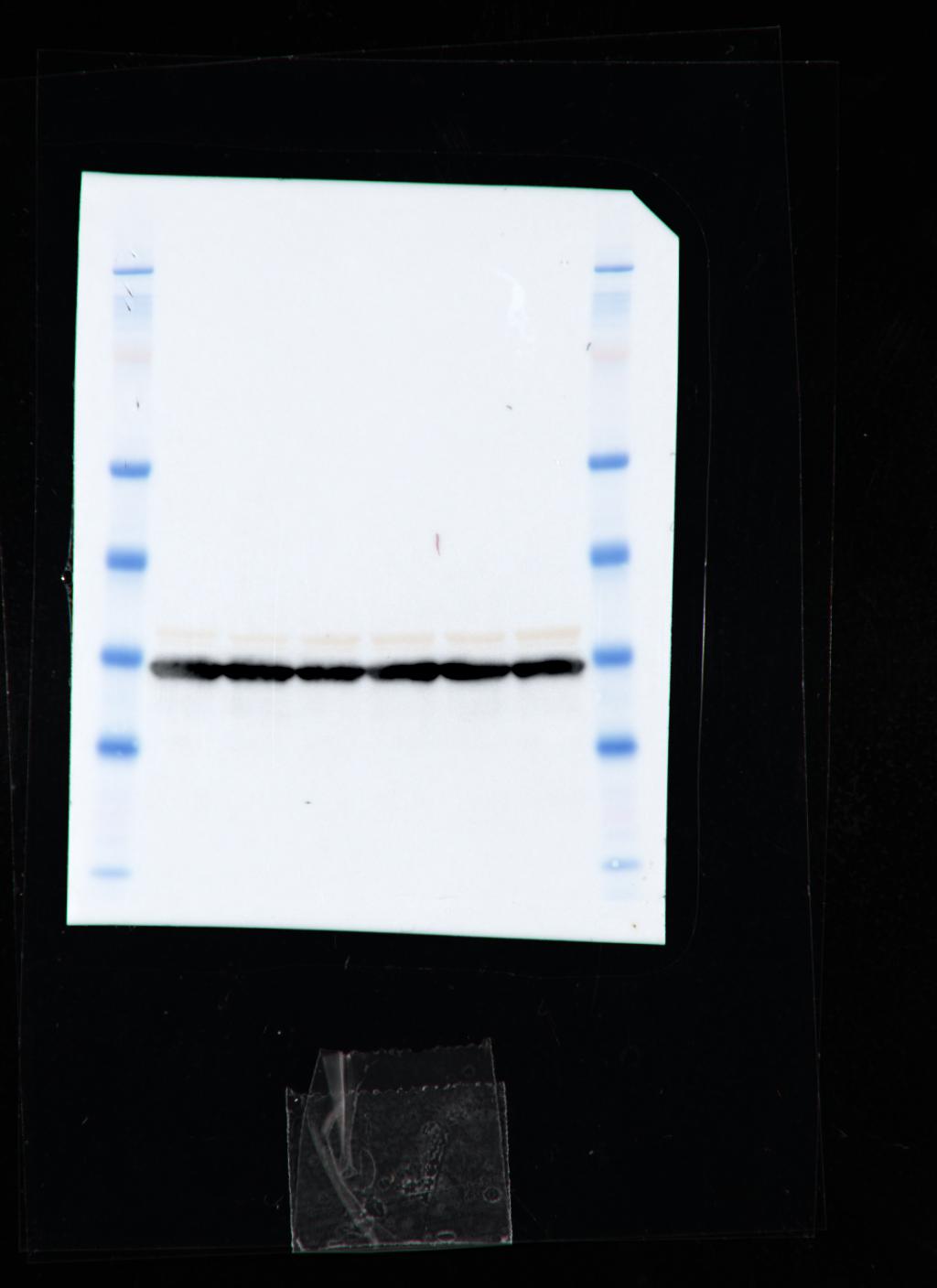
**

**Supplementary Western Blot GAPDH**

Raw Western blot of which a cropped version is used in figures 3 and 4.

Lane 1: protein ladder marker

Lane 2: day 0 of differentiation sample

Lane 3: day 4 of differentiation sample

Lane 4: day 7 of differentiation sample (used as untreated control in figure 4)

Lane 5: day 7 of differentiation, 72h incubated with 0.5 µM ryuvidine

Lane 6: day 7 of differentiation, 72h incubated with 10 ng/ml TNFα

Lane 7: day 7 of differentiation, 72h incubated with 10 ng/ml TNFα and 0.5 µM ryuvidine

Lane 8: protein ladder marker


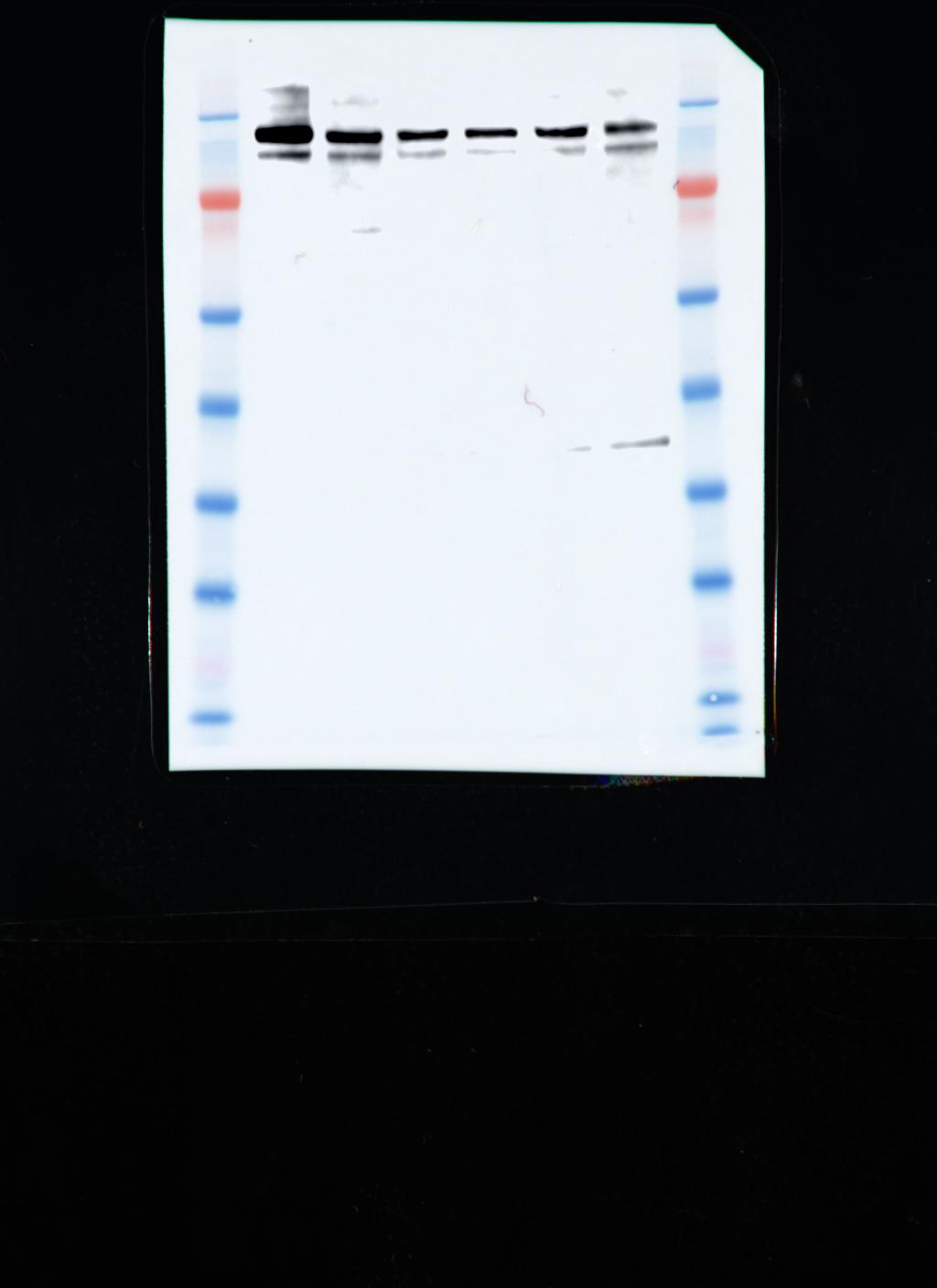


**Supplementary Western Blot KDM5A**

Raw Western blot of which a cropped version is used in figures 3 and 4.
